# Disseminated cysticercosis in a child: a rare occurrence presenting as meningitis

**DOI:** 10.1186/1471-2334-14-S3-P49

**Published:** 2014-05-27

**Authors:** A Arunkumar, I Vivian Praveen, HV Prajwala, Asha Benakappa

**Affiliations:** 1 Department of Pediatrics, Bangalore Medical College and Research Institute, Bangalore, India

## Background

Neurocysticercosis, a parasitic disease is one of the commonest causes of focal convulsions. Disseminated cysticercosis (DCC) is amongst its rarest manifestations. Fewer than 50 cases have been described worldwide with less than 10 in children. We report a 9 year old female child with the disease presenting as meningitis.

## Case report

A 9 year old female child presented with fever, headache, myalgia for 15 days, vomiting and altered sensorium for 2 days. She was well nourished, disoriented, febrile, had meningeal signs. CSF examination was unremarkable. CT brain was normal. She was treated as viral meningo-encephalitis and improved. Her subsequent admission with similar complaints showed grossly elevated CSF proteins and lymphocytes, MRI brain showed solitary ring enhancing lesion in brain and treated as tuberculous meningitis with tuberculoma. She did not show improvement, presented back with multiple nodules all over the body. Ultra-sonogram revealed them to be subcutaneous and muscular cysticercoses. MRI showed disseminated cysticercoses involving all areas of brain. She was immunocompetent. She was started on oral prednisolone followed by albendazole for a month. She showed improvement, nodules subsided, had convulsions in follow-up visits, and is on regular anticonvulsants.

## Conclusion

The main features of DCC include intractable epilepsy, dementia, muscular enlargement, subcutaneous nodules and absence of focal neurological signs. Our case is remarkable because of combination of disseminated neuro and myo-cysticercosis and meningitis. Short duration of complaints with widespread dissemination in an immunocompetent child necessitates further understanding on the disease process and warrants suspicion of cysticercal meningitis in endemic areas.

